# A tale of shifting relations: East Asian summer and winter monsoon variability during the Holocene

**DOI:** 10.1038/s41598-021-85444-7

**Published:** 2021-03-25

**Authors:** Stefanie Kaboth-Bahr, André Bahr, Christian Zeeden, Kweku A. Yamoah, Mahjoor Ahmad Lone, Chih-Kai Chuang, Ludvig Löwemark, Kuo-Yen Wei

**Affiliations:** 1 Institute of Geosciences, University of Potsdam, Potsdam-Golm, Germany; 2 Institute of Earth Sciences, Heidelberg University, Heidelberg, Germany; 3 LIAG, Leibniz Institute for Applied Geophysics, Hannover, Germany; 4 School of Geography, Earth and Environmental Sciences, University of Birmingham, Edgbaston, UK; 5 High-Precision Mass Spectrometry and Environment Change Laboratory, Department of Geosciences, National Taiwan University, Taipei, Taiwan ROC; 6 Department of Geosciences, National Taiwan University, Taipei City, Taiwan ROC

**Keywords:** Environmental sciences, Ocean sciences, Solid Earth sciences

## Abstract

Understanding the dynamics between the East Asian summer (EASM) and winter monsoon (EAWM) is needed to predict their variability under future global warming scenarios. Here, we investigate the relationship between EASM and EAWM as well as the mechanisms driving their variability during the last 10,000 years by stacking marine and terrestrial (non-speleothem) proxy records from the East Asian realm. This provides a regional and proxy independent signal for both monsoonal systems. The respective signal was subsequently analysed using a linear regression model. We find that the phase relationship between EASM and EAWM is not time-constant and significantly depends on orbital configuration changes. In addition, changes in the Atlantic Meridional Overturning circulation, Arctic sea-ice coverage, El Niño-Southern Oscillation and Sun Spot numbers contributed to millennial scale changes in the EASM and EAWM during the Holocene. We also argue that the bulk signal of monsoonal activity captured by the stacked non-speleothem proxy records supports the previously argued bias of speleothem climatic archives to moisture source changes and/or seasonality.

## Introduction

**Figure 1:**
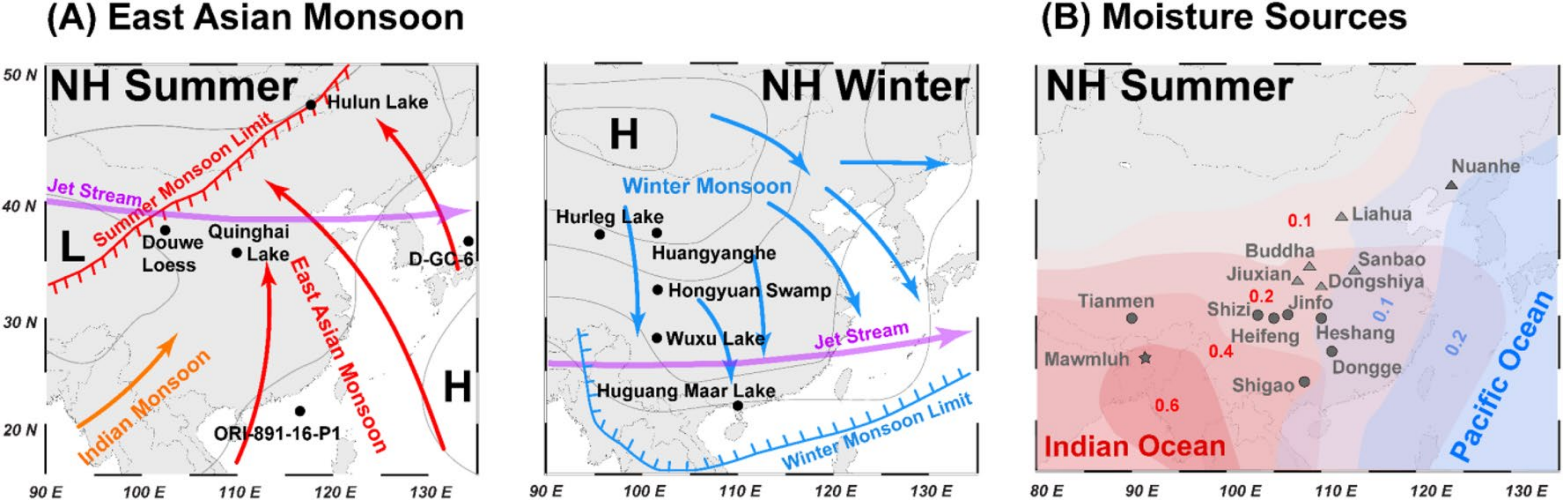
East Asian summer (EASM) and winter monsoon (EAWM) purview and sites used for this study. (**A**) Atmospheric conditions during North Hemisphere summer (EASM). Northward propagating warm and moisture charged air masses are indicated by red arrows (left panel); Atmospheric conditions during North Hemisphere winter (EAWM). Southward trending cold air outbreaks are highlighted by blue arrows (right panel); (**B**) Moisture sources of East Asia during North Hemisphere summer (EASM)^45^. Numbers constitute relative contribution of the Indian Ocean (red) or Pacific Ocean (blue) moisture source to total amount of precipitation over East Asia. Sites used for the EASM and EAWM stack are marked by black dots in (**A**) and (**B**), respectively (Tables S1, S2). Purple arrow indicates position of the Jetstream. Location of the speleothem reference sites for the northern and southern Chinese speleothem stacks are marked by grey triangles and dots, respectively. Mawmluh speleothem cave site is marked with a grey star.

The East Asian monsoon system (EAM), which includes both the summer and winter Asian monsoons (EASM and EAWM), affects the hydroclimate variability of large parts of South-East Asia (Fig. 1A and B)^1,2^. During late spring to summer, the large thermal contrast between the warm Asian continent and the adjacent colder oceans leads to moist-laden air from the Indic and Pacific Ocean to be traversed into East Asia. This initiates EASM precipitation from southern China to the Korean Peninsula and Japan^3^. During the onset of EAWM, the thermal land–ocean contrast reverses, strengthening the high-pressure system above Siberia. This causes cold air outbreaks emanating from the Siberian High during winter periods reaching latitudes as low as 20° N^4,5^.

On interannual to decadal time scales, both the EASM and EAWM, show strong spatio-temporal variability. This variability has been primarily linked to changes in the El Niño-Southern Oscillation (ENSO) and in association also the Pacific Walker Circulation (PWC). The effect of El Niño (La Niña) on the EASM is typically propagated through changes in the convective activities over the western Pacific affecting the Hadley circulation^6^. El Niño events are characterized by a strong sea surface temperature (SST) warming in the eastern Pacific Ocean thereby causing anomalous subsidence over Borneo and Indonesia. The effects of ENSO on summer monsoonal precipitation over Asia is instable and varies regionally^7,8^. Generally, observational data and numerical models have argued an overall decrease in summer precipitation during El Niño, and vice versa for La Niña conditions^7,8^. In case of the EAWM, El Niño (La Niña) events have been argued to trigger the development of an anomalous lower-tropospheric anticyclonic (cyclonic) circulation around the Philippines via Gill-type Rossby wave responses in winter that leads to a weak (strong) EAWM^6,9^. In addition, supra-regional forcing mechanisms such as changes in the Atlantic Meridional Overturning Circulation (AMOC)^10,11^, Arctic sea-ice coverage^12,13^, sunspot activity^14,15^, and global greenhouse gas fluctuations [i.e. carbon dioxide (*p*CO_2_) and methane (*p*CH_4_)^16,17^], have all been linked to EAM variability on multiple time scales. In particular, the relationship between AMOC and EASM has gathered substantial interest over the previous decade, as the 8.2 kyr cooling event, probably caused by a meltwater outburst that slowed the AMOC^18,19^, is proposed to have triggered a strong centennial-long reduction of EASM. In this scenario, the reduction of the AMOC would enforce a decreased northward meridional heat transport which leads to cooling over North America and the formation of an anomalous high-pressure cell^20^. The high pressure induces stronger northeast winds across the equator and strengthens the upwelling of cold water in the eastern equatorial Pacific Ocean. As a result, the equatorial Walker Circulation is strengthened on the expense of the latitudinal Hadley Cell which ultimately causes a weakening of the EASM^20^. Of great public interest is also the effect of *p*CO_2_ on EAM variability because the anthropogenic induced rising *p*CO_2_ is argued to promote enhancement of EASM strength while the EAWM will likely be suppressed, causing winter warming and droughts^21^.

Despite the relevance of the EAM variability, the dynamic relationship between EASM and EAWM on millennial to orbital timescales remain ambiguous. This could be attributed to the contradicting or inconclusive findings based on observational data, model simulations, and variable sensitivities of available hydroclimatic proxies^21^. This particularly regards the question whether speleothem stable oxygen (δ^18^O) reconstructions from Chinese caves, often considered a pristine archive of EASM variability, are reflecting predominantly changes in the moisture source rather than precipitation amounts^22–24^.

Hence, this study aims to extract a robust EASM and EAWM signal for the last 10,000 years by combining available marine and terrestrial (non-speleothem) proxy records from the East Asian realm (Fig. 1A and B). The geographical wide spread selected sites and proxies provide an unbiased view on East Asian monsoon variability independent of specific regions, proxies and individual age models. We argue that the extracted signal, for both monsoonal systems, provides a more reliable basis to test the influence of potential driving mechanisms such as *p*CO_2_ on the spatio-temporal EASM and EAWM variability. To decipher the response of EASM and EAWM to these driving forces we conducted a linear regression model assuming that the contribution of each driver is linear to the EASM and EAWM signal during the course of the Holocene. Lastly, the reconstructed EASM and EAWM signal was also used to test the sensitivity of speleothem recorded climate change during the Holocene.

## Results

### Proxy record stacking

**Figure 2:**
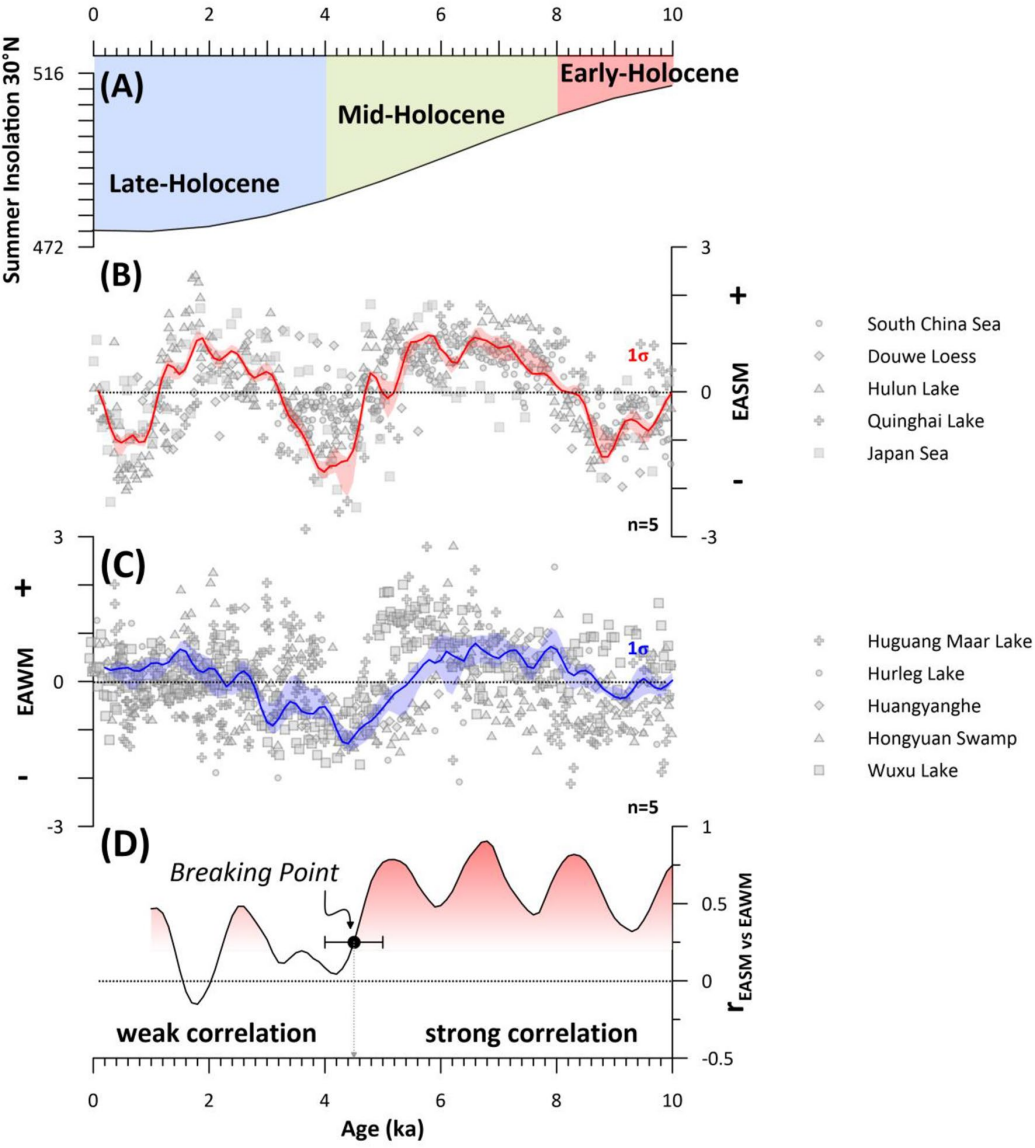
East Asian summer (EASM) and winter (EAWM) monsoon stacks. (**A**) 21st June (summer) insolation for 30° N^25^; The 2.5% and 97.5% (1 σ) uncertainty intervals for the stacks are shown as shaded (**B**) red (EASM) and (**C**) blue (EAWM) areas. The detrended original data sets used for each stack are marked by grey icons according to the Figure legend. (**D**) moving Spearman correlation coefficient (r) with window size = 1 between EASM and EAWM stacks. Shading indicates strong correlation (red) to weak correlation (white). The identified break point at 4.5 ± 0.5 kyr is indicated by a black dot with error bars.

The EASM stack (see “Material and methods” section; Fig. 2B) shows a clear millennial-scale pacing during the last ~ 10 kyr. Intensified (positive values) EASM coincides with the time interval between 8.2–4.7 and 3.3–1.2 kyr. In contrast, weakened EASM (negative values) coincide with three phases centered at ~ 9, ~ 4 and ~ 0.6 ka, respectively. The EAWM stack (see “Material and methods” section; Fig. 2C) also shows millennial-scale variability during the last ~ 10 ka. Intensified (positive values) EAWM coincide with the time interval between 8.2 and 5.6 kyr, while a long-term trend of increasing EAWM strength can be observed starting from ~ 3.5 ka until 0.4 ka. In contrast, a weakened EASM (negative values) coincide with two phases from 9.4–8.2 kyrs to 5.6–3.5 kyrs, respectively.

### Spearman correlation coefficient (r) and break point analysis

The correlation coefficient between EASM and EAWM shows a distinct weakening from the Early to Mid-Holocene towards the late Holocene (see “Material and methods” section; Fig. 2D). Between ~ 10–4.5 kyr the r value ranges between 0.3 and 0.8. After ~ 4.5 kyr the r value is generally below 0.3 with two maxima visible around ~ 2.6 and 1.0 kyr where r = 0.5. The calculated break point (see “Material and methods” section) is centred at 4.5 kyr with the 1σ error of ± 0.5 kyr.

### Linear model simulation

To test the relationship between our EASM and EAWM signals and their driving mechanisms (Tabs. 1, S1–S3) we assumed in a first order approximation a linear relationship. The regression was significant for both EASM and EAWM. The R^2^ for of the model fit for EASM and EAWM is 0.53 and 0.51, respectively. This suggests that more than half of the data variance can be explained with the applied linear model.

**Table 1:** Linear model simulation of East Asian summer monsoon (EASM; Table S1) and winter monsoon (EAWM; Table S2) versus selected driving mechanisms (Table S3).

	EASM		EAWM	
	t value	*p* value	t value	*p* value
AMOC	4.454	2.61e^−05^	1.605	0.112306
CH_4_	0.185	0.85337	2.416	0.017882
CO_2_	− 2.918	0.00453	− 2.314	0.023161
ENSO	2.769	0.00694	3.494	0.000765
ICE	3.282	0.00151	3.080	0.002808
SS	− 0.602	0.54891	− 4.162	7.67e^−05^

The t value is the regression coefficient divided by its standard error. The size of the t-value for each mechanism is a measure of the effect that mechanism is having on EASM or EAWM variability, and its (positive or negative) sign provides the direction of the effect. The *p* value denotes the significance level for the relationship between the mechanisms, and EASM as well as EAWM variability. See “Material and methods” section for detail description the linear regression model.

The results for the EASM model (Table 1) show that AMOC and EASM are significantly positive correlated. Significant albeit weaker linear relationships also exist between EASM and ENSO as well as Arctic sea ice coverage. A negative significant relationship can be observed between EASM and CO_2_. Neither sun spot numbers nor CH_4_ exhibit a significant linear relationship with the EASM.

The results of the EAWM model implies a positive and significant relationship between EAWM to ENSO, sun spot numbers and Arctic sea ice coverage (Table 1). Similar to the EASM, we also find a significant negative relationship between EAWM and CO_2_. The EAWM is seemingly not significantly linearly related to AMOC.

## Discussion

The developed EASM and EAWM stacks highlight distinct millennial-scale features (Fig. 2B,C). During the Early Holocene to Mid-Holocene both EASM and EAWM show synchronous periods of weakening (~ 9 kyrs), strengthening (~ 6–8 kyrs) and again weakening (~ 4 kyrs). This synchronous behaviour seemingly changes after ~ 4 kyrs as both monsoonal systems diverge: while the EAWM is characterized by a steady ~ 3 kyr increase in strength from the mid-Holocene towards the modern era, the EASM shows no long-term trend during the late Holocene but instead another strong reduction at ~ 0.6 ka, which is not mimicked by the EAWM. This change in relationship between EASM and EAWM is also clearly depicted in the temporal evolution of the correlation coefficient between both stacks (Fig. 2D) which demonstrates that both monsoonal systems were strongly correlated during the Early to Mid-Holocene and subsequently disconnected with experiencing partially opposing trends during the Late Holocene.

The here presented evolution of both monsoonal systems during the Holocene stands in contrast to the previously argued persistent out-of-phase behaviour between EASM and EAWM for this time period^21,26,27^. In this scenario, a weakening of the EASM is generally considered to go hand in hand with a strengthened EAWM, and vice versa. However, our findings align with modelling results that argue that changes in the orbital configuration, which define the seasonal and latitudinal distribution of incoming solar radiation, lead to a variable relationship between EASM and EAWM during the geological past^28^. These findings imply that our observed divergence in the relationship between EASM and EAWM during the Holocene could have been related to the progressive reduction of insolation in the low-latitudes at the same time (Fig. 2).

During increased insolation conditions the seasonality of the Northern Hemisphere is increased which leads to much warmer summer, and colder winter than compared to decreased insolation conditions of the late Holocene^25^. The enhanced surface cooling during winter of the Early to Mid-Holocene leads to a strengthening of the Siberian High and enhances the thermal gradient between the Asian continent and the warmer adjacent oceans. The resulting thermal pressure gradient is directed from land to the ocean, and facilitates a strengthening of the EAWM. During summer the increased insolation levels cause significant warming over the land in the mid- and high latitudes of the Northern Hemisphere^28^. The surface warming triggers an enhanced rising of warm air masses over the Asian continent which, in turn, invokes a strong transport of water laden air masses from the Oceans into the interior of the Asian continent, and thus contributes to an enhanced EASM. Hence, an in-phase strengthening of the EASM and EAWM as depicted by our stacks during the Early to Mid-Holocene is plausible if the respective orbital configuration is considered.

With the passing of an insolation threshold at ∼4.5 ka the in-phase relationship between EASM and EAWM seemingly changed leading to an out-of-phase behaviour of both monsoonal system under decreasing boreal summer insolation of the late Holocene (Fig. 2). The divergence of EASM and EAWM during the mid- to late Holocene indicates that their variability during the last ~ 4 kyr was not predominantly driven by insolation forcing. Instead, we hypothesize that the reduced seasonality under decreased boreal summer insolation as well as the reduced annual net-insolation of the Northern Hemisphere^25^ increased the sensitivity of EASM and EAWM to other drivers such as AMOC and ENSO variability, changes in *p*CO_2_ and *p*CH_4_, sun spot numbers as well as Arctic sea ice variability. To ground-truth this hypothesis, we conducted a linear modelling approach testing the correlation of the EASM and EAWM stacks to their proposed driving mechanisms.

**Figure 3:**
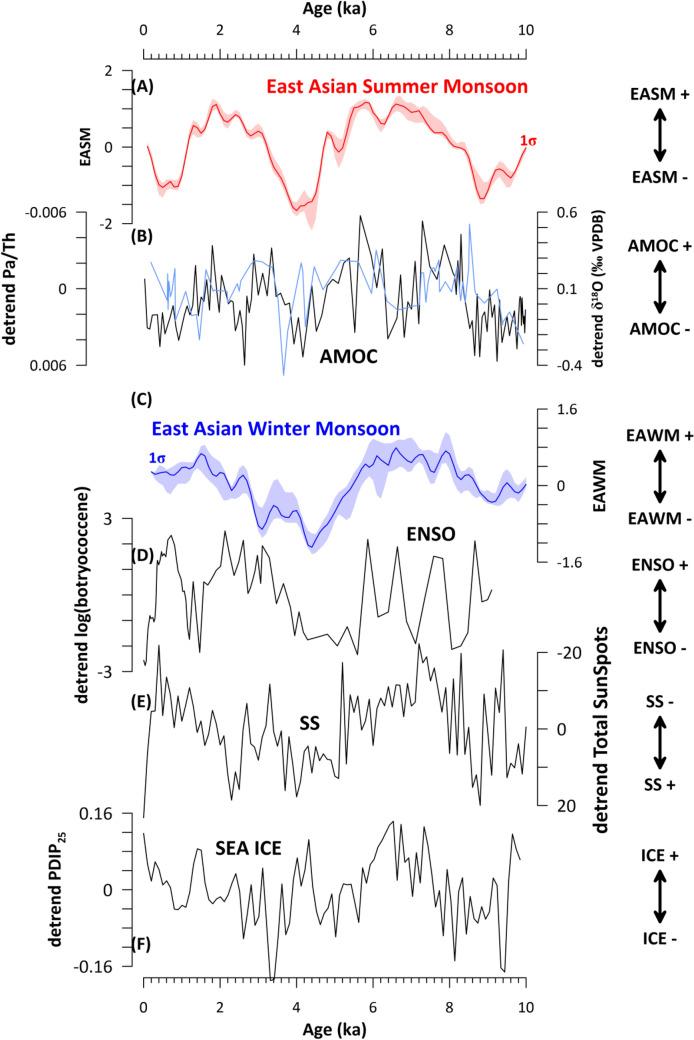
Comparison of East Asian summer (EASM) and winter (EAWM) monsoon stacks to their dominant driver. (**A**) East Asia summer monsoon (EASM) stack with 1 σ uncertainty (this study); (**B**) Proxies for Atlantic Meridional Overturning Circulation (AMOC) based on detrended Δδ^18^O_w_ of Site RAPiD-12-1K^30^ (black line) and detrended Pa/Th concentrations of Site ODP1063^29^ (blue line); (**C**) East Asia winter monsoon (EAWM) stack with 1σ uncertainty (this study); (**D**) Proxy for El Niño-Southern Oscillation (ENSO) based on detrended log(botryococcene concentration in μg/g) derived from El Junco^31^; (**E**) detrended Total Number of Sun Spots (SS)^32^; (**F**) Proxy for Arctic sea ice coverage based on the concentrations of brassicasterol (PBIP_25_) concentration derived from Site ARAB2B-1A^33^.

Our model results show that in particular the weak phases of the EASM at ~ 9 ka, ~ 4 ka and ~ 0.6 ka are linked to changes in AMOC variability (Table 1, Fig. 3A and B). This significant positive correlation is in line with simulation data^20^, as well as previous suggestions from the Late Pleistocene^11^. Interestingly, the strongest reduction of EASM occurs at ~ 4 ka and might thus be intrinsic linked to 4.2 kyr event which has been argued to relate to the strongest AMOC reduction during the Holocene^29^. In contrast, the 8.2 kyr-event did not leave a discernible fingerprint in our EASM stack (Fig. 3) which is in line new findings that imply the meltwater outburst associated with 8.2-kyr event did not result in a pronounced AMOC reduction^29^. Instead, we find a strong EASM reduction at ~ 9 ka which predates the 8.2 kyr-event.

The EAWM, on the other hand, is predominantly correlated to changes in ENSO, SS and SEA ICE (Fig. 3C–F; Table 1). The significant negative relationship between EAWM and SS found in our analysis is supported by previous proxy studies which suggest that the EAWM will increase in intensity when the number of sunspots decreases (Fig. 3)^14,34^. In fact, the impact of solar irradiance changes on the EAWM has also been suggested on model results for the last 600 years^15^. The connection between EAWM and SS (Fig. 3) during the Holocene is most likely amplified by sea ice variability, which we find the EAWM is significantly positively correlated to. The latter has been shown to be strongly sensitive to SS changes on millennial-time scales^33^. In this scenario, an increase of sea ice in the Barents Sea region under low solar irradiance leads to enhanced albedo, together with a strengthening of the Siberian High and a southward migration of the East Asian trough. This process induces cooling in mid-latitude Asia and strengthens the EAWM, and acts opposing during during low sea-ice cover conditions^35,36^. A different mediator for the coupling between EAWM and SS has been proposed via AMOC forcing^15^. However, the apparent lack of a significant correlation between EAWM and AMOC (Fig. 3) during the Holocene rather suggests that this mechanism played a minor role.

We also find a significant positive correlation between EAWM and ENSO (Fig. 3) which at first sight opposes the negative correlation suggested from previous studies^6,9^. However, recent findings suggest that the impact of ENSO on the EAWM differs with the geographical position of the SST anomaly within the equatorial Pacific Ocean^37^. If the SST warming associated with the El Niño is located in the central rather than the eastern Pacific Ocean it promotes cooling in both the eastern and western equatorial Pacific Ocean. This in turn leads to an anomalous sinking motion over the eastern Pacific Ocean, which enforces the prevalence of dominant northerly or northeasterly wind over southern China associated with strong EAWM^37^. This line of argumentation would align with our results and suggests that the geographical position of the western Pacific Ocean warming/cooling might have played a much more prominent role in shaping EAWM variability than previously suggested.

Interestingly, EASM and EAWM both also show significant negative correlations to *p*CO_2_ (Table 1). This implies that EASM and EAWM would weaken under increased *p*CO_2_. The negative correlation between EAWM and *p*CO_2_ is in line with observational and model results suggesting a weakening of the EAWM under global warming (increased *p*CO_2_) conditions^38,39^. Yet, the relationship between EASM strength and *p*CO_2_ remains ambiguous with some model studies suggesting that a continued rise in *p*CO_2_ most likely would lead to enhanced monsoonal precipitation over East Asia in the future (positive correlation)^40^ whereas the analysis of observational data from the last three decades imply a weakening of the summer monsoonal system (negative correlation)^41^. The significant negative relationship we find between the EASM and *p*CO_2_ during the Holocene would suggest that the proposed increase of EASM strength under future global warming scenarios might not have had an analogue during the last 10,000 years.

**Figure 4:**
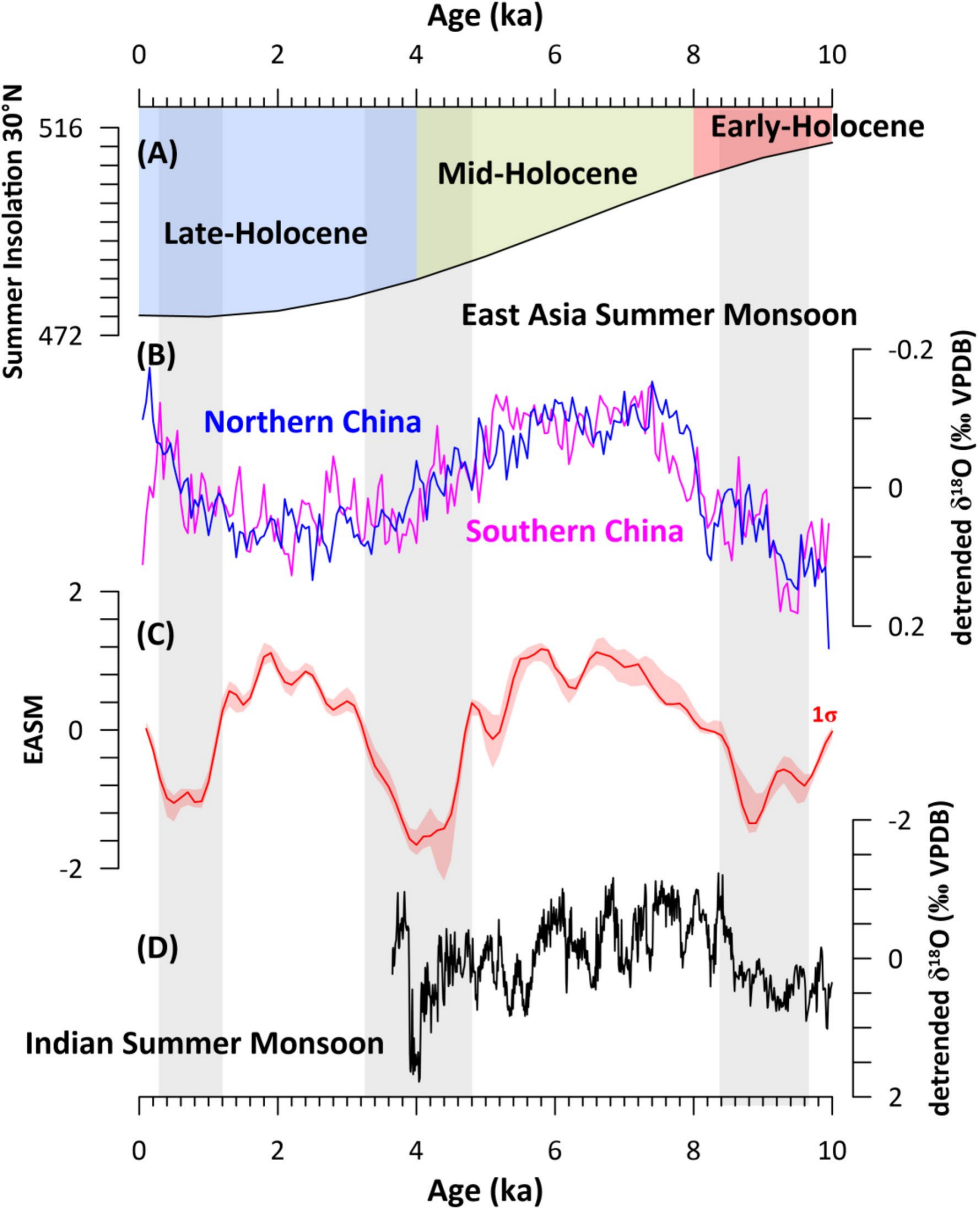
Comparison between the East Asian summer monsoon stack (EASM), and Chinese and Indian speleothem δ^18^O records. (**A**) 21st June (summer) insolation for 30°N^25^; (**B**) Detrended δ^18^O_calcite_ records from Northern and Southern China^46^ (Fig. 4B); (**C**) East Asia summer monsoon (EASM) stack with 1σ uncertainty (this study); (**D**) Detrended δ^18^O_calcite_ record of stalagmite KM-A from the Indian Mawmluh cave site^43^. Grey shaded columns designate weak EASM phases according to our EASM stack.

Lastly, we compared our EASM stack to δ^18^O speleothem records from East Asia to assess a potential moisture source-related bias on speleothem records from the EASM domain during the Holocene (Fig. 4). For the comparison we used the previously published northern and southern China δ^18^O speleothem stacks to avoid location-specific biases of the speleothem data (due to i.e. seepage path, karst fissure water, convective cave ventilation and kinetic fractionation^42^). The comparison shows that the millennial-scale variability captured in our EASM stack fits well with the Chinese δ^18^O speleothem evolution during the Early to Mid-Holocene (high insolation levels) but distinctively diverges from it during the Mid- to Late Holocene (Fig. 4). This might be viewed as an indication for the insensitivity of non-speleothem records to EASM changes, and argues against their application as proposed in this study. However, the comparison of our EASM stack to the Mawmluh δ^18^O speleothem record^43^, a recorder for the Indian Summer Monsoon (ISM), yields a close correlation also during the Mid-Holocene (Fig. 4). These observations imply that during the Early to Mid-Holocene, and under increased insolation conditions, Chinese δ^18^O speleothem records captured EASM amount changes in-phase with our EASM stack. On the other hand, that the signal divergences between both records during the Mid- to Late Holocene could indicate that Chinese δ^18^O speleothems potentially record a change in moisture source rather than precipitation amount. This is in line with previous suggestions that argue that the Chinese speleothem δ^18^O signal is sensitive to the moisture source effect^22,23^ or to changes in the relative proportion of the moisture sources on annual rainfall^24^. In contrast, we argue that our EASM stack responds predominantly to the precipitation amount, as the proxies used for the stack capture e.g., vegetation and sea surface temperature changes or terrigenous run-off to the ocean, and thus might be more integrative for the entire summer season. In fact, the good signal correlation between our EASM stack and the ISM signal (Fig. 4), could indicate that under P_max_ conditions the prevalence of Indian Ocean sourced moisture increased its dominance across China over the moisture sourced from the West Pacific, thus mimicking modern conditions^44,45^ (Fig. 1B). Based on these results we argue that the Holocene EASM variability based on Chinese speleothem δ^18^O do not necessarily represent the entire EASM history but rather a regional proportion of it. This could also explain the differences in long-term variability between non-speleothem and speleothem climatic proxies in East Asia.

In summary, we constructed robust indices for EASM and EAWM variability during the Holocene which provide a supra-regional, proxy-independent reflection of monsoonal strength. We find that during the Early to Mid-Holocene both, EASM and EAWM, are tightly coupled and strengthened, whereas during the mid-to late Holocene the evolution of EASM and EAWM seems to diverge. Based on these results, we argue that this shift in relationship is intrinsically linked to low-latitude insolation changes that passed a crucial insolation threshold at ~ 4.5 kyr. The insolation reduction most likely also heightened the sensitivity of both monsoonal systems to other low and high latitude driving mechanisms such as ENSO, AMOC, and Arctic sea ice conditions. Interestingly, the sensitivity of the EAWM to greenhouse gas changes during the Holocene closely mimics modern observations. However, we note that the EASM variability at the same time reacts inversely to the proposed *p*CO_2_ forcing under future global warming scenarios. Hence, the proposed sensitivity of the EASM to rising *p*CO_2_ levels in the next 100 years might not have had an analogue in the last 10,000 years. We also find that the observed EASM variability differs markedly from the proposed EASM evolution during the Holocene based on Chinese δ^18^O speleothem records but instead aligns well with the Indian δ^18^O speleothem signal. The latter observation critically underlines the debated sensitivity of δ^18^O from speleothems to seasonal moisture source changes whereas non-speleothem proxy records, as used in this study, might be more robust to capture seasonal changes in absolute precipitation amount during the Holocene.

## Material and methods

To analyze the spatio-temporal variability of the EASM and EAWM during the Holocene we combined a total of ten proxy records which have been distinctively assigned to either EASM (n = 5; Table S1, Figs. 1, S1) or EAWM (n = 5; Table S2, Figs. 1, S2) variability. For our synthesis we adopted the initial chronology for each proxy record as well as the respective proxy interpretation (see Table S1).

We combined the individual EASM and EAWM records by linear resampling (0.1 kyr step size) and detrending each individual proxy record before applying a low pass Taner filter with a cut-off frequency of 2 and a roll-off-rate of 10^10^. These steps were implemented to adjust for the differences in sample resolution and account for respective age uncertainties between the different proxy records, as well as remove the long-term insolation trend inherent to the selected records. The stacking was realized with an iteration loop with N = 1000 iterations. For this we used the *astrochron* package implemented in the software R^47,48^. To test the robustness of the stacking we also generated randomized EAWM stacks where one contributing data set was alternating removed from the stacking and compared it to the EAWM stack over all available data sets and its 1σ envelope (Fig. S3, Table S2) The R script for the stacking is attached in the *supplementary information*.

To constrain the relationship between EASM and EAWM with applied a moving Spearman correlation coefficient (r) with the window size = 1 using the *astrochron* package implemented in the software R (Fig. 2)^47,48^. Spearman's correlation coefficient measures the strength and direction of association between two time series, Additionally, we calculated potential break points of the correlation coefficient series (Fig. 2) derived from a fitting of a linear regression model to the coefficient series as implemented in the *strucchange* package in R^4,47^. The algorithm tests deviations from stability in a classical linear regression model with a pre-set of maximum m = 5 breakpoints. The integrated optimization algorithm provides the optimized location of the breaking points as well as their 1σ confidence level (Fig. 2). The F-statistics of the calculated break point is also calculated (Fig. S4). Both R scripts for the moving Spearman correlation and the break point analysis available in the *supplementary information*.

As a next step, we test the relationship between EASM and EAWM and a suite of selected driving mechanisms/reference datasets (*p*CO_2_, *p*CH_4_, AMOC; SEA ICE, ENSO, and SS; see Table S3). For this we applied a linear model using the ’lm’ function implemented in the software R^48^. This method statistically tests whether a linear relationship exists between an input signal (EASM or EAWM) and different driving reference signals, significance tests do not account for serially correlated data and should be treated with care. We used the calculated EASM and EAWM stacks as input signal. The R script for the linear model simulation is attached in the *supplementary information*.

## Supplementary Information

Supplementary Information

## Data Availability

All original data sets used for the stacking are accessible through their respective cited references. The stacking results are online available via PANGAEA.de.
