# Supplementary material for: A tale of shifting relations: East Asian summer and winter monsoon variability during the Holocene
Source: Sci Rep. 2021 Mar 25;11:6938. doi: 10.1038/s41598-021-85444-7 (PMC7994397; doi:10.1038/s41598-021-85444-7)
Supplement: Supplementary file 1 — Supplementary Information [file 41598_2021_85444_MOESM1_ESM.docx]

*Supplementary Information* for

A tale of shifting relations: East Asian summer and winter monsoon variability during the Holocene

Stefanie Kaboth-Bahr^1*^, André Bahr^2^, Christian Zeeden^3^, Kweku Yamoaha^4^, Mahjoor Lone^5^, Chih-Kai Chuang^5^, Ludvig Löwemark^5^, Kou-Yen Wei^5^

^1^ University of Potsdam, Institute of Geosciences, Potsdam-Golm, Germany

^2^ University Heidelberg, Institute of Earth Sciences, Heidelberg, Germany

^3^ LIAG, Leibniz Institute for Applied Geophysics, Hannover, Germany

^4^ University of Birmingham, School of Geography, Earth and Environmental Sciences, Edgbaston, UK.

^5^ National Taiwan University, Department of Geosciences, Taipei City, Taiwan ROC

*Corresponding author: Stefanie Kaboth-Bahr (kabothbahr@uni-potsdam.de)

**Content**

Figure S1

Figure S2

Figure S3

Figure S4

Table S1

Table S2

Table S3

R Code “Stacking”

R Code “Moving Correlation”

R Code “Breaking Points”

R Code “Linear Model”

References

**Table S1:** Overview of sites and proxy records used for the development of the East Asian summer monsoon (EASM) stack.

| Site | Region | Lat (N) | Lon (E) | # AMS ^14^C tie points | Proxy | Interpretation | Reference |
| --- | --- | --- | --- | --- | --- | --- | --- |
| HL-06 | Hulun Lake | 48°25’ | 117°20’ | 13 | Pollen-based annual precipitation (P_a_) | High P_a_: stronger EASM; low P_a_: weaker EASM | ^1^ |
| D-GC-6 | Japan Sea | 37°3’ | 134°42’ | 12 | electron spin resonance signal intensity (ESR) | High ESR: increased dust input from Gobit desert signals earlier seasonal northward progression of the Westerly Jet which correlates with stronger EASM.  Low ESR: increased dust input from Taklamakan desert signals more southward progression of the Westerly Jet which correlates with weaker EASM. | ^2^ |
| LQDP05-1Fs | Quinhai Lake | 36°48’ | 100°08’ | 21 | UK^37^-  based summer temperature (T_S_) | High summer T: stronger EASM; low summer T: weaker EASM | ^3^ |
| loess/soil sequence | Douwe Loess | 35°25’ | 101°57’ | 13 | pedogenic susceptibility-based annual precipitation (P_a_) | High P_a_: stronger EASM; low P_a_: weaker EASM | ^4^ |
| ORI-891-16-P1 | South China Sea | 19° 33’ | 116° 6’ | 4 | log(Ti/Ca) | High log(Ti/Ca): high terrigenous influx due to strong EASM; low log(Ti/Ca): decreased terrigenous influx due to weaker EASM | Kaboth-Bahr et al., *submitted* |

**Figure S1.** Overview of the original proxy records for the East Asian Summer Monsoon stack (EASM; see Tab. S1 for more details on site location, age model, proxy record used and reference).

**
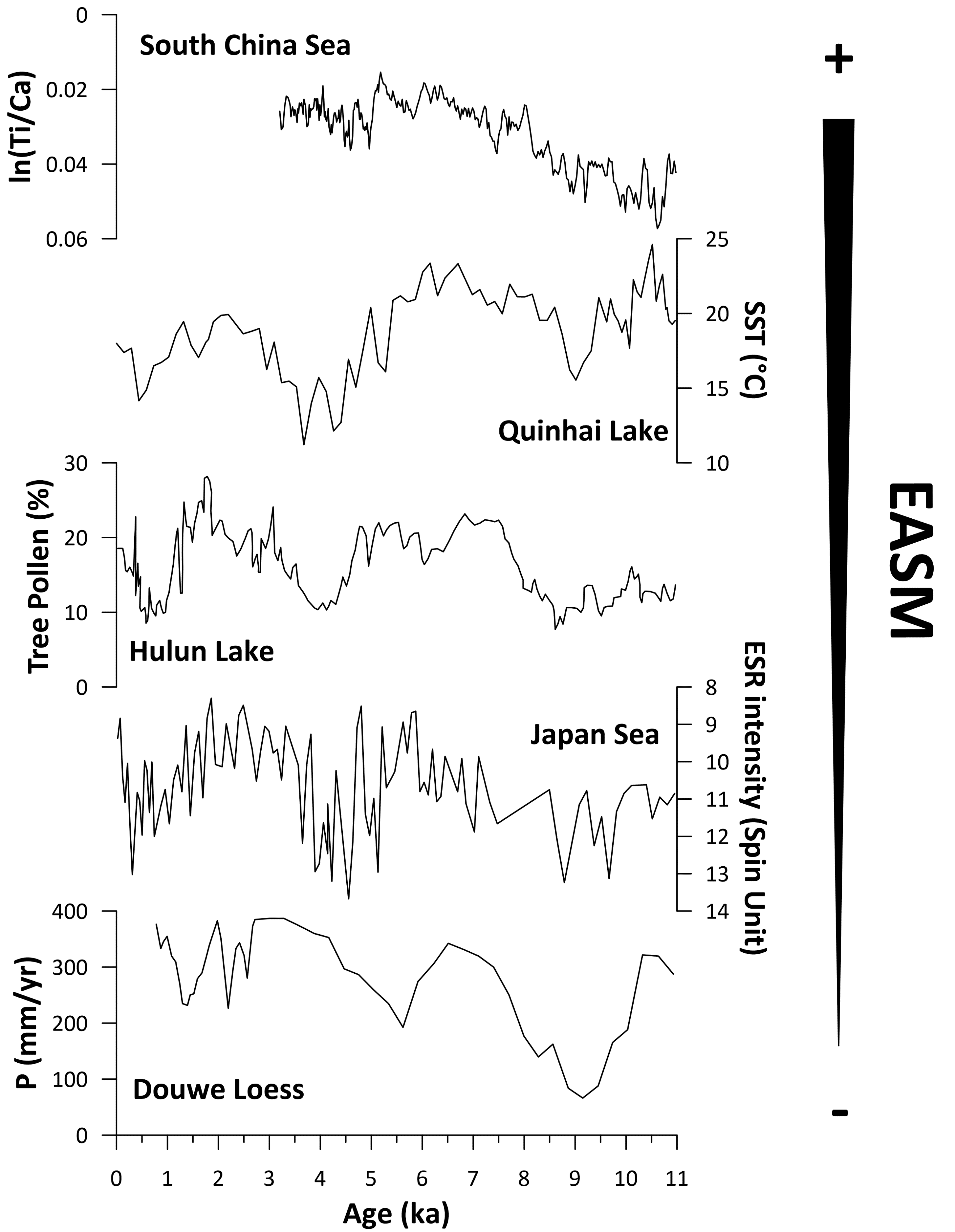
**

**Table S2**: Overview of sites and proxy records used for the development of the East Asian winter monsoon (EAWM) stack.

| Site | Region | Lat (N) | Lon (E) | # AMS ^14^C tie points | Proxy | Interpretation | Reference |
| --- | --- | --- | --- | --- | --- | --- | --- |
| HUG-A- HUG-G | Huguang Maar Lake | 21°9’ | 110°17’ | 9 | AG/CS ratio  ratio (*A. granulata*/*C. stelligera*) | High AG/CS: strong EAWM; low AG/CS: weaker EAWM | ^5^ |
| HL05-2 | Hurleg Lake | 37°17’ | 96°54’ | 7 | A/C ratio (*Artemisia/Chenopodiaceae*) | High A/C ratio: stronger EAWM; low A/C ratio: weaker EAWM | ^6^ |
| Section a | Huangyanghe | 37°25’ | 102°36’ | 7 | TOC | High TOC: weaker EAWM; low TOC: stronger EAWM | ^7^ |
| HY2A | Hongyuan Swamp | 32°46’ | 102°31’ | 11 | Dust flux | High dust flux: stronger EAWM; low dust flux: weaker EAWM | ^8^ |
| UWITEC | Wuxu Lake | 29°9’ | 101°24’ | 16 | PCA2 from pollen assemblage | High PCA2: weak EAWM.  Low PCA2: stronger EAWM. | ^9^ |

**Figure S2.** Overview of the original proxy records for the East Asian Summer Monsoon (EAWM; see Tab. S2 for more details on site location, age model, proxy record used and reference).

**
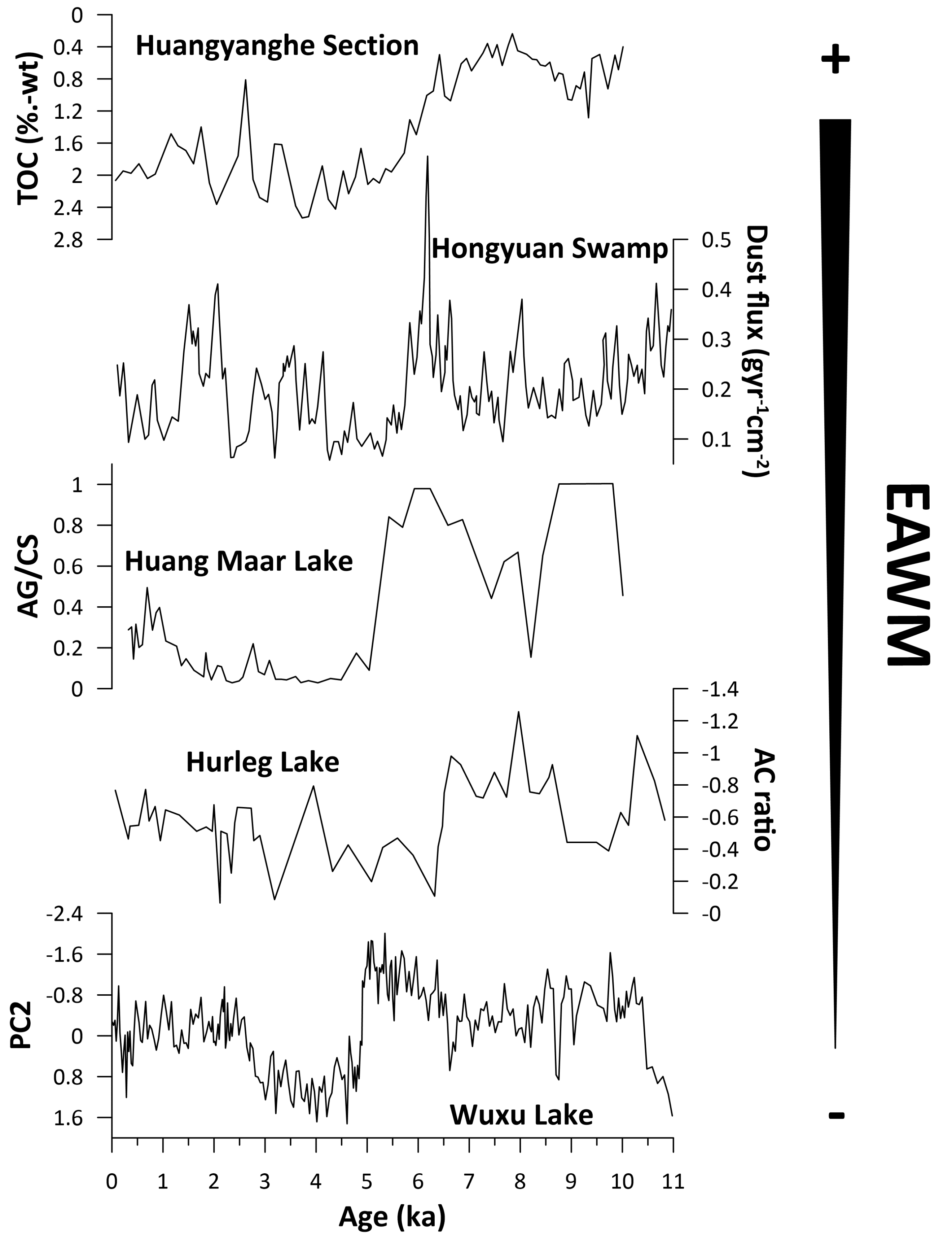
**

**Table S3**: Overview of selected proxy records that represent proposed driving mechanisms of the East Asian summer (EASM) and winter monsoon (EAWM).

| ID | Site | Lat (N) | Lon (E) | Proxy | Interpretation | Reference |
| --- | --- | --- | --- | --- | --- | --- |
| ENSO | El Junco | 00°18’ | 91°00’ | Log(Botryococcene concentration) | High Log(Botryococcene concentration): increased El Niño frequency; low Log(Botryococcene concentration): decreased El Niño frequency | ^10^ |
| SS | INTCAL98 | Global |  | Δ^14^C from tree rings | High total number of sunspots: increased sun activity; low total numbers of sunspots: decreased sun activity | ^11^ |
| CH_4_ | EPICA | -75°06' | 123°21' | *p*CH_4_ | High *p*CH_4_: increased atmospheric methane concentration; low *p*CH_4_: decreased atmospheric methane concentration | ^12^ |
| CO_2_ | EPICA | -75°06' | 123°21' | *p*CO_2_ | High *p*CO_2_: increased atmospheric methane concentration; low *p*CO_2_: decreased atmospheric methane concentration | ^12^ |
| AMOC | RAPiD-12-1K | 62°05’ | -17°49’ | Δδ^18^O | High Δδ^18^O: strong AMOC; low Δδ^18^O: weak AMOC | ^13^ |
| ICE | ARAB2B-1A | 73°37’ | -166°30 | PBIP25 | High PBIP25: increased western Arctic sea ice cover; low PBIP25: decreased western Arctic sea ice cover | ^14^ |

**Figure S3.** Robustness test of the East Asian Winter Monsoon (EAWM) stack. EAWM stack with all five data sets (EAWM_full_; black line; as stated in the main text); red line denotes the 1σ envelope of the EAWM_full_; EAWM_a_ to EAWM_e_ denotes stacks where one of the five available data sets was randomly removed from the stacking.


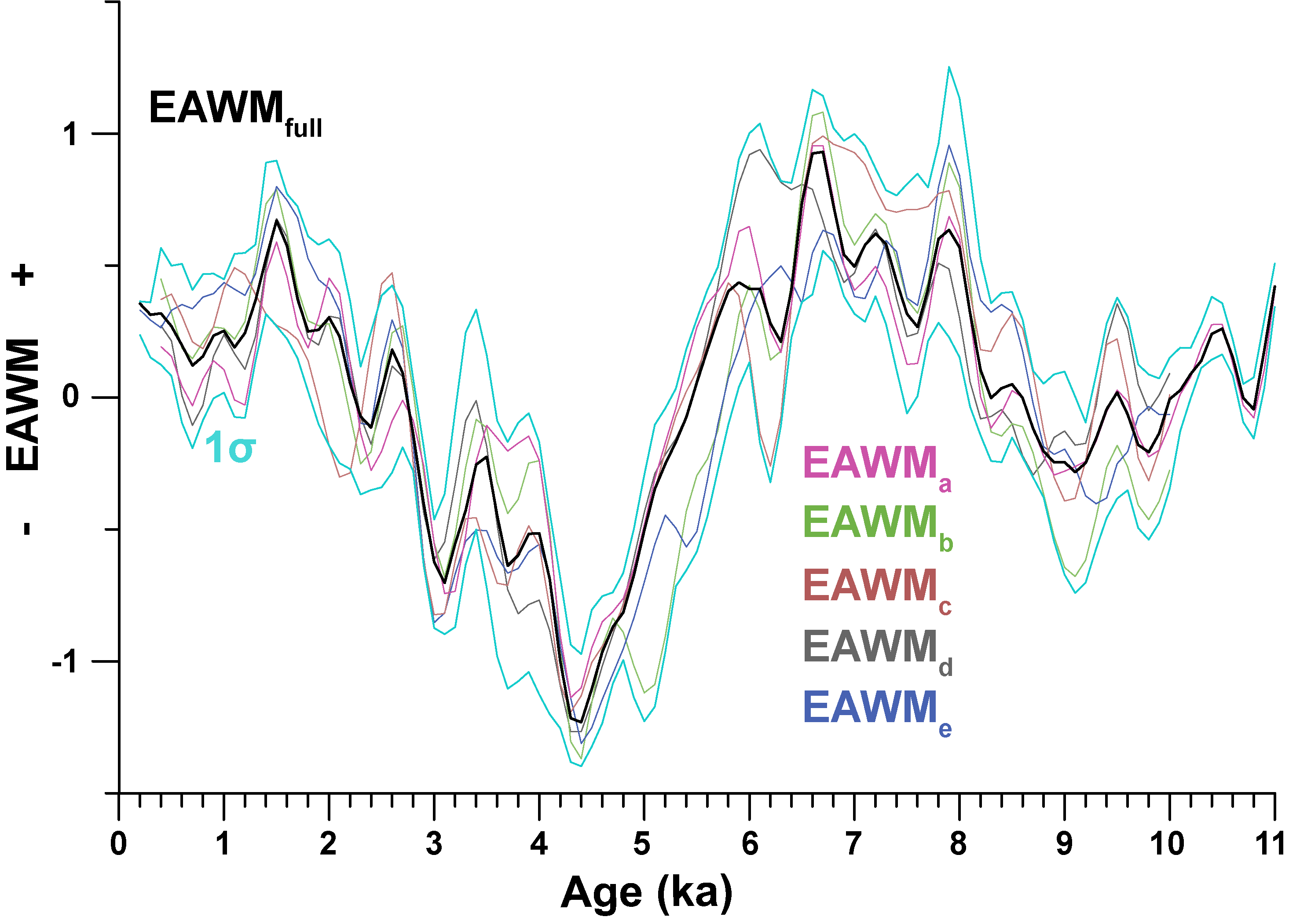


**Figure S4.** F-statistic of the breaking point analysis based on the windowed correlation coefficient between EASM and EAWM. The identified dominant break point at 4.5 kyr is indicated by a dotted line. Critical F value threshold is denoted by a red solid line.


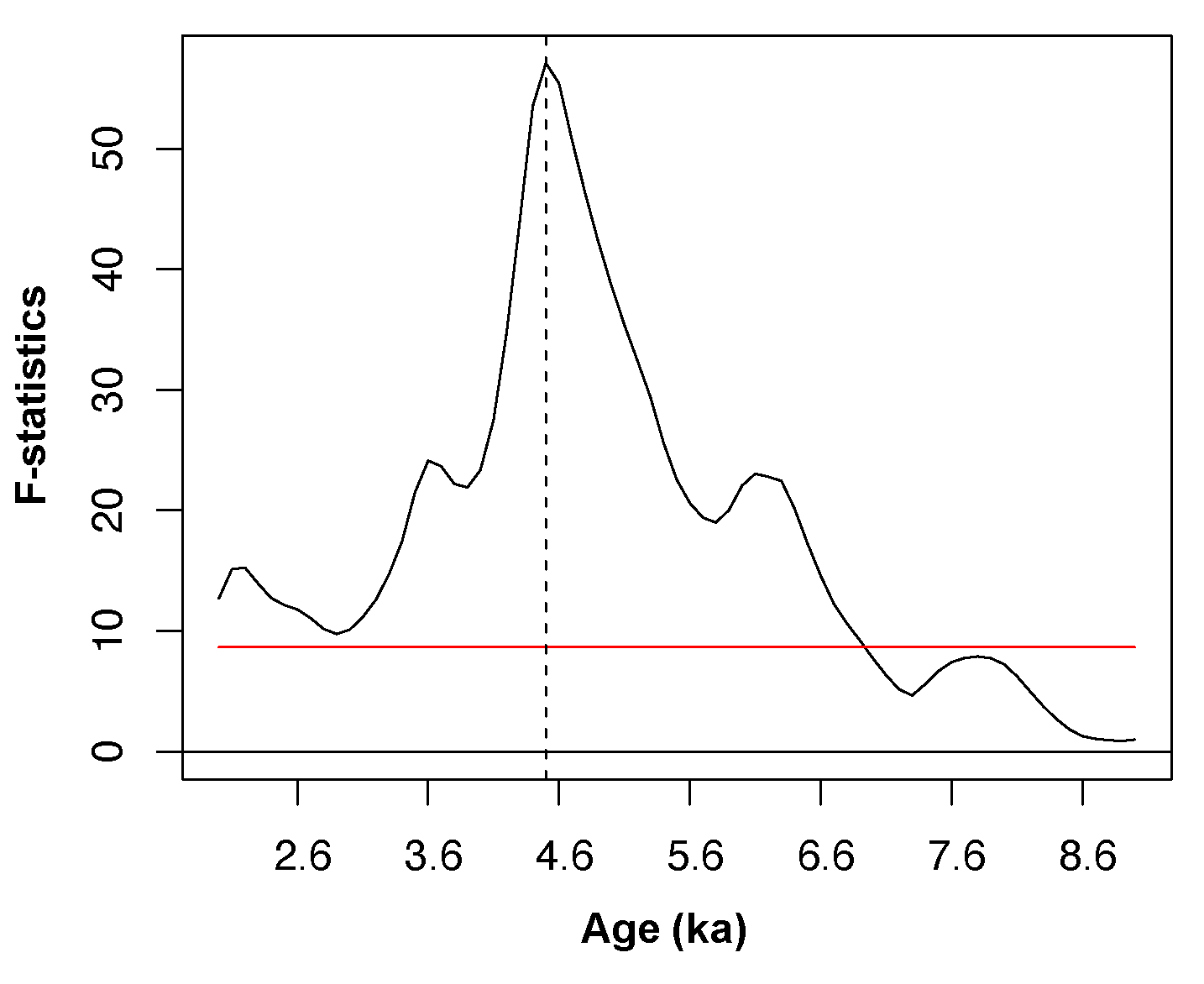


**R code “Stacking”**

# script to construct a Asian Monsoon stacks

# scripted by C. Zeeden (christian.zeeden[at]leibniz-liag.de), February 2018

# load libraries, which must be installed

library(astrochron)

library(readxl)

library(birk)

# set up working directory - will need adjustment on every computer

# this is an EXAMPLE, and will need adjustment, depending on where your data is stored

setwd('/Users/kaboth-bahr/Documents/Paper/South China Sea')

# load datasets from excel file

# dataset 1: South China Sea (EASM)

# (dataset this study, not initialy used for stacking)

Data1 <- read_excel("Data for Stacks.xlsx", sheet = "South China Sea")

#View(Data1)

Data1 <- cb(Data1[,2], -Data1[,4])

plot(Data1, type="l")

# dataset2: Quinhai Lake (EASM)

Data2 <- read_excel("Data for Stacks.xlsx", sheet = "Quinhai Lake")

#View(Data2)

Data2 <- cb(Data2[,2], Data2[,4])

plot(Data2, type="l")

# dataset3: Hulun Lake (EASM)

Data3 <- read_excel("Data for Stacks.xlsx", sheet = "Hulun Lake")

#View(Data3)

Data3 <- cb(Data3[,1], Data3[,2])

plot(Data3, type="l")

# dataset4: Japan Sea (EASM)

Data4 <- read_excel("Data for Stacks.xlsx", sheet = "Japan Sea")

#View(Data4)

Data4 <- cb(Data4[,1], -Data4[,2])

plot(Data4, type="l")

# dataset5: Duowe Loess (EASM)

Data5 <- read_excel("Data for Stacks.xlsx", sheet = "Duowe Loess")

#View(Data5)

Data5 <- cb(Data5[,1], -Data5[,3])

plot(Data5, type="l")

# dataset6: Huanyanghe Section (EAWM)

Data6 <- read_excel("Data for Stacks.xlsx", sheet = "Huangyanghe section")

#View(Data6)

Data6 <- cb(Data6[,1], -Data6[,2])

plot(Data6, type="l")

# dataset7: Hongyuan Swamp (EAWM)

Data7 <- read_excel("Data for Stacks.xlsx", sheet = "Hongyuan Swamp")

#View(Data7)

Data7 <- cb(Data7[,1], -Data7[,3])

plot(Data7, type="l")

# dataset8: Huang Maar Lake (EAWM)

Data8 <- read_excel("Data for Stacks.xlsx", sheet = "Huang Maar Lake")

#View(Data8)

Data8 <- cb(Data8[,5], -Data8[,7])

plot(Data8, type="l")

# dataset9: Hurleg Lake (EAWM)

Data9 <- read_excel("Data for Stacks.xlsx", sheet = "Hurleg Lake")

#View(Data9)

Data9 <- cb(Data9[,1], -Data9[,3])

plot(Data9, type="l")

# dataset10: Wuxu Lake (EAWM)

Data10 <- read_excel("Data for Stacks.xlsx", sheet = "Wuxu Lake")

#View(Data10)

Data10 <- cb(Data10[,1], -Data10[,2])

plot(Data10, type="l")

####################################

####################################

# place selected data in list 'ID'

# exaple here: EAWM

ID <-list (Data1, Data2, Data3, Data4, Data5)

####################################

####################################

# stacking:

# concept:

# 1) resample data (all 5 datasets) on age model (use 80% of data)

# 2) linearly interpolate data

# 3) cut to common length (of shortest dataset)

# 4) linear detrending, smooth data using a low-pass Taner filter, standardize

# 5) stack data

# - repeat many times

# 6) obtain stack and uncertainty for stack

# define functions 'minf' and 'maxf' to obtain the minimum/maximum value (age) of 1st column

minf <- function(x) {min(na.omit(x)[,1])}

maxf <- function(x) {max(x[,1])}

# define function to store results of the 'linterp' function as matrix

# resampling resolution will be set to 0.1 (can be easily be adjusted here - but it will need an adjustment of the length of data storing variables)

# !define interpolation HERE

Linterp <- function(x){as.matrix(linterp(x, dt=.1, genplot=F, start=0))}

# define function to store results of the 'detrend' function as matrix, omit empty rows

Detrend <- function(x){as.matrix(detrend(na.omit(x)), genplot=F)}

# define function to store results of the 'taner' function as matrix, omit empty rows

# ! Taner filter settings to be set HERE

Taner <- function(x){as.matrix(taner(x, fhigh=2, roll=10^10, genplot=F, verbose=F))}

#set number of simulations

Nsim <- 1000

# set percentage to be resampled

ResPercentage <- 80

# initiate matrix for storing results (stacks)

# NOT a general case, but a matrix for a specific case

StackRes <- matrix(NA,200,(Nsim+1))

StackRes[,1] <- seq(from=0, to=19.9, by=0.1)

for (i in 1:Nsim)

{

# step1:

# resample datasets

S1 <- sample(ID, size=ceiling(length(ID)*ResPercentage/100))

#initiate new matrix for storing resamled results

S2 <- S1

#resample elements (age, monsoon proxy data) from S1, save as S2

for (l in 1: length (S1))

{

S2[[l]] <- S1[[l]][sort(sample( x = dim(S1[[l]])[1],

size=floor(dim(S1[[l]])*ResPercentage/100)[1],

replace=F)),]

}

# step 2

# linear interpolation of data

# apply to list

S3 <- lapply(S2,

Linterp)

# step 3

# cut dataset to lengths where at least 2 datasets are present for stacking

Min <- sort(sapply(S3, minf))[2]

Max <- sort(sapply(S3, maxf), decreasing = TRUE)[2]

# cut list to length

for (k in 1:length(S3))

{

S3[[k]] <- S3[[k]][which(S3[[k]][,1]==Min):max(which(S3[[k]][,1]<=Max)),]

}

# step 4

# Detrend all datastes in list

S4 <- lapply(

S3,

Detrend)

# standardize all datastes in list

S5 <- lapply(S4,

s,

verbose=F)

# step 4

# low-pass filter data to focus on longer term trends

S6 <- lapply(S5,

Taner)

# plot for check (optional)

#plot(S6[[1]], type="l")

#lines(S6[[2]])

#lines(S6[[3]])

#lines(S6[[4]])

# place all data in matrix

Mx <- matrix(NA, length((0:(Max*10))), length(S6)+1)

Mx[,1] <- StackRes[0:(Max*10+1),1]+.1

for (j in 1:length(S6))

{

Mx[(min(S6[[j]][,1])*10):(max(S6[[j]][,1])*10),(j+1)] <- S6[[j]][,2]

}

Mx <- na.omit(Mx)

# step 5: stacking of data

# (finally): compile stack by taking the average/mean of all datasets

# save results to variable

StackRes[(which(StackRes[,1]==Mx[1,1])):(which.closest(StackRes[,1], max(Mx[,1]))),(i+1)] <- rowMeans(Mx[,-1], na.rm=TRUE)

# output counter to command line (optional)

print(i)

}

# step 6

# compile stack and uncertainty as derived from resampling

Stack1 <- cb(StackRes[,1],

t(apply(StackRes[,2:Nsim],

1,

quantile,

probs=c(0.025, 0.16, 0.5, 0.84, 0.975),

na.rm=T)

))

# plot mean Stack (optional)

dev.off()

plot(Stack1[[1]], Stack1[[4]], type="l", xlab="Time", ylab="Stack", xlim=c(0,12), lwd=2)

lines(Stack1[[1]], Stack1[[3]])

lines(Stack1[[1]], Stack1[[5]])

# export result

# getwd() will display the working directory where results are stored

write.csv(Stack1, file="EASM_full.csv")

# end script

**R code Moving Spearman Correlation**

# script Breaking point analysis

# scripted by S. Kaboth-Bahr (kabothbahr[at]uni-potsdam.de), Jan2021

# load libraries, which must be installed

library(astrochron)

library(readxl)

# set up wd

setwd('/Users/kaboth-bahr/Desktop')

#load data from an Excel file

dat <- read_excel("corr_V1.xlsx")

#moving Spearman correlation with window size = 1

mwCor(dat,win=1 ,cormethod=2,output=T,pl=1,genplot=T,verbose=T)

#export result

getwd() #will display the working directory where results are stored

write.csv(mwcorr, file = 'mwcorr.csv') # export as .csv

# end script

**R code Breaking point analysis**

# script Breaking point analysis

# scripted by S. Kaboth-Bahr (kabothbahr[at]uni-potsdam.de), Jan2021

library(readxl)

library(forecast)

library(tseries)

library(strucchange)

library(nonlinearTseries)

library(sandwich)

# set up wd

setwd('/Users/kaboth-bahr/Desktop')

## load the data from an Excel file

data <- read_excel("mwcor_result.xlsx")

## store data as time series objects

mwcorr <- as.ts(data$corr, start=c(1.5, 1), end=c(10.2, 1), frequency=0.1)

### F statistics to indicate how many breakpints

fs.mwcorr <- Fstats(mwcorr ~ 1)

plot(fs.mwcorr)

breakpoints(fs.mwcorr)

lines(breakpoints(fs.mwcorr))

# calculate breakpoints with set maximal number

bp.mwcorr <- breakpoints(mwcorr ~ 1, breaks = 3)

summary(bp.mwcorr)

## plot the graph with breakdates and their confidence intervals

plot(bp.mwcorr)

plot(mwcorr)

lines(bp.mwcorr)

ci.mwcorr <- confint(bp.mwcorr)

ci.mwcorr

lines(ci.mwcorr)

# end script

**R code “Linear Model”**

# script Linear Model

# scripted by S. Kaboth-Bahr (kabothbahr[at]uni-potsdam.de), May2020

# install libraries, which must be installed

library(astrochron)

library(readxl)

# read data to R

var <- read_excel("/Users/kaboth-bahr/Documents/Paper/South China Sea/lm3_ice rev.xlsx")

var <- as.data.frame(var)

# extract individual datasets

amoc<- cb(var[,1], var[,2])

easm <- cb(var[,1], var[,3])

ss <- cb(var[,1], var[,4])

ch4 <- cb(var[,1], var[,5])

co2 <- cb(var[,1], var[,6])

enso <- cb(var[,1], var[,7])

eawm <- cb(var[,1], var[,8])

ice <- cb(var[,1], var[,9])

# low-pass filter datasets

amocT <- taner(amoc, fhigh=2, roll=10^10, genplot=F, verbose=F)

ssT <- taner(ss, fhigh=2, roll=10^10, genplot=F, verbose=F)

ch4T <- taner(ch4, fhigh=2, roll=10^10, genplot=F, verbose=F)

co2T <- taner(co2, fhigh=2, roll=10^10, genplot=F, verbose=F)

ensoT <- taner(enso, fhigh=2, roll=10^10, genplot=F, verbose=F)

iceT <- taner(ice, fhigh=2, roll=10^10, genplot=F, verbose=F)

#linear model set-up & output

#amoc2 <- amocT[,2]^2

lmvar <- lm(easm[,2] ~ amocT[,2]+ssT[,2]+ch4T[,2]+co2T[,2]+ensoT[,2]+iceT[,2])

#lmvar <- lm(eawm[,2] ~ amocT[,2]+ssT[,2]+ch4T[,2]+co2T[,2]+ensoT[,2]+iceT[,2])

lmvar

summary(lmvar)

plot(eawm, col="red", type="l")

lines(cb(eawm[,1], lmvar$fitted.values))

# optional’: plot results

#plot(amocT[,2], easm[,2], pch=16, ylab = "EASM", cex.lab = 1.3, col = "red" )

#abline(lm(easm[,2] ~ amocT[,2]), col = "blue")

# optional: export data (to do so remove ‘#’ at the beginning of lines)

#write.table(amocT,file = "/Users/kaboth-bahr/Documents/Paper/South China #Sea/amocT.txt", append = TRUE)

#write.table(ssT,file = "/Users/kaboth-bahr/Documents/Paper/South China Sea/ssT.txt", #append = TRUE)

# end script

**References**

1. Wen, R. *et al*. Holocene precipitation and temperature variations in the East Asian monsoonal margin from pollen data from Hulun Lake in northeastern Inner Mongolia, China. *Boreas* **39**, 262–272 (2010).

2. Nagashima, K., Tada, R. & Toyoda, S. Westerly jet‐East Asian summer monsoon connection during the Holocene. *Geochemistry, Geophys. Geosystems* **14**, 5041–5053 (2013).

3. Hou, J. *et al*. Large Holocene summer temperature oscillations and impact on the peopling of the northeastern Tibetan Plateau. *Geophys. Res. Lett*. **43**, 1323–1330 (2016).

4. Maher, B. A. & Hu, M. A high-resolution record of Holocene rainfall variations from the western Chinese Loess Plateau: antiphase behaviour of the African/Indian and East Asian summer monsoons. *The Holocene* **16**, 309–319 (2006).

5. Wang, L. *et al*. The East Asian winter monsoon over the last 15,000 years: Its links to high-latitudes and tropical climate systems and complex correlation to the summer monsoon. *Quat. Sci. Rev*. **32**, 131–142 (2012).

6. Zhao, Y., Yu, Z., Chen, F., Ito, E. & Zhao, C. Holocene vegetation and climate history at Hurleg Lake in the Qaidam Basin, northwest China. Rev. *Palaeobot. Palynol*. **145**, 275–288 (2007).

7. Li, Y. & Morill, C. A Holocene East Asian winter monsoon record at the southern edge of the Gobi Desert and its comparison with a transient simulation. *Clim. Dyn*. **45**, 1219–1234 (2015).

8. Yu, X., Zhou, W., Zhao, L. & Kang, Z. Different patterns of changes in the Asian summer and winter monsoons on the eastern Tibetan Plateau during the Holocene. *The Holocene* **21**, 1031–1036 (2011).

9. Zhang, E., Wang, Y., Sun, W. & Shen, J. Holocene Asian monsoon evolution revealed by a pollen record from an alpine lake on the southeastern margin of the Qinghai-Tibetan Plateau, China. *Clim. Past Discuss*. **12**, 415–427 (2015).

10. Zhang, Z., Leduc, G. & Sachs, J. P. El Niño evolution during the Holocene revealed by a biomarker rain gauge in the Galápagos Islands. *Earth Planet. Sci. Lett*. **404**, 420–434 (2014).

11. Solanki, S. K., Usoskin, I. G., Kromer, B., Schüsseler, M. & Beer, J. Unusual activity of the Sun during recent decades compared to the previous 11,000 years. *Nature* **431**, 1084–1087 (2004).

12. Monnin, E. et al. Atmospheric CO2 concentrations over the last glacial termination. *Science* **291**, 112–114 (2001).

13. Thornalley, D. J. R., Elderfield, H. & McCave, I. N. Holocene oscillations in temperature and salinity of the surface subpolar North Atlantic. *Nature* **457**, 711–714 (2009).

14. Stein, R. *et al.* Holocene variability in sea ice cover, primary production, and Pacific-Water inflow and climate change in the Chukchi and East Siberian Seas (Arctic Ocean). *J. Quat. Sci.* **32**, 362–379 (2017).
